# Analysing the Launch of COVID-19 Vaccine National Rollouts: Nine Case Studies

**DOI:** 10.3390/epidemiologia2040036

**Published:** 2021-10-23

**Authors:** John Gannon, Razieh Azari, Marta Lomazzi, Bettina Borisch

**Affiliations:** World Federation of Public Health Associations, 1202 Geneva, Switzerland; razieh.azari@wfpha.org (R.A.); marta.lomazzi@wfpha.org (M.L.); bettina.borisch@unige.ch (B.B.)

**Keywords:** COVID-19, vaccination, procurement, communication, distribution

## Abstract

In late 2020 and early 2021, with the eagerly anticipated regulatory approval of vaccines against SARS-CoV-2, the urgent global effort to inoculate populations against this devastating virus was underway. These case studies examine the early stages of COVID-19 vaccine rollouts across nine regions from around the world (Brazil, India, Indonesia, Ireland, Israel, Nigeria, Taiwan, United Kingdom and United States). By evaluating and comparing different approaches used to immunize against a novel pathogen, it is possible to learn a great deal about which methods were successful, and in which areas strategies can be improved. This information is applicable to the ongoing global vaccination against this virus, as well as in the event of future pandemics. Research was conducted by following and tracking the progress of vaccine rollouts in the nine regions, using published clinical trials, government documents and news reports as sources of data. Results relate to the proportion of populations that had received at least one COVID-19 dose by 28 February 2021. Outcomes are discussed in the context of three key pillars integral to all immunization programs: procurement of vaccines, communication with the public and distribution of doses to individuals.

## 1. Introduction

COVID-19 is the most seismic public health crisis of our lifetimes. Mass immunization offers the best chance to contain the pandemic, but it presents a fiercely complex challenge for governments, healthcare systems and populations. This article explores the vaccination strategies and policies that were implemented in different regions of the world to protect citizens against the virus. Under analysis are the preparations and performances of Brazil, India, Indonesia, Ireland, Israel, Nigeria, Taiwan, United Kingdom and United States, in 2020 and over the first two months of 2021, as their COVID-19 vaccine rollouts were launched.

The motivation for this research was to compare strategies taken by a range of governments and the results of those actions. This would provide an opportunity to appreciate which aspects were done well and to identify mistakes that were made, or challenges that hindered progress. Lessons have been learned from global vaccination campaigns against diseases such as smallpox, polio, influenza, diphtheria and myriad others, and this experience is continually utilized. It is imperative that we scrutinize global efforts to vaccinate against SARS-CoV-2 so that we can improve our understanding of how best to combat infectious agents. Knowledge about which strategies are most effective will be essential for the ongoing immunization against COVID-19 and its variants. When we face the inevitable next pandemic caused by a novel pathogen, it will also be useful to retrospectively examine steps taken by different countries and regions in 2020 and 2021, so that we have a robust evidence base on which to guide our actions. We will want to learn in particular from this experience how to rapidly procure millions of doses, clearly communicate rollout plans with populations and maximize compliance and efficiently distribute new vaccines on a massive scale to protect against a previously unknown micro-organism.

The nine regions were selected for the research to provide a broad global geographical scope (five continents are represented, including the most populous countries in North America, South America and Africa and second and third most populous in Asia). There is also a diversity of economic prosperity represented in the case studies: Ireland, Israel, Taiwan, UK and US are high-income, Brazil is upper-middle income, and Indonesia, India and Nigeria are lower-middle income nations. Three of the countries (India, UK and US) notably played a key role in vaccine development and manufacturing. Israel’s world-beating rapid start to the rollout and Taiwan’s tremendous record at keeping COVID-19 case numbers down prior to the introduction of vaccines, accounted for their addition. Ireland was included as a representative of the European Union, and as it is the home of this article’s first author.

The ‘Results’ section of this article features analyses of the early stages of the vaccine rollouts of these nine regions. In each case, progress in acquiring vaccine doses and organizing the immunization drives is discussed, as well as the magnitude of the virus’ impact at that time. The case studies differ significantly in the timing, quantity, costs and manufacturer choice in relation to vaccine procurement and this is covered in the text. Efforts to address hesitancy and optimize communication, prioritization systems, and logistics and distribution aspects are addressed. In the ‘Discussion’, comparisons are made between the nine regions in three key areas: procurement, communication and distribution. The ‘Conclusions’ highlight the features that are integral to effective national rollouts, and also explore the issue of global inequity relating to COVID-19 vaccination.

## 2. Materials and Methods

Vaccine candidate clinical trials and published data were accessed to provide information about the various available vaccines, their mechanisms of action, and the evidence supporting their efficacy and safety. Government websites and published policies available online were analyzed to establish the central plans and steps taken in preparing for and coordinating national vaccine rollouts. Finally, health statistics websites, news articles and reports and opinion surveys were used to monitor the ongoing progress, updates, setbacks and unique features of COVID-19 vaccination campaigns across these nine regions.

## 3. Results

**Figure 1:**
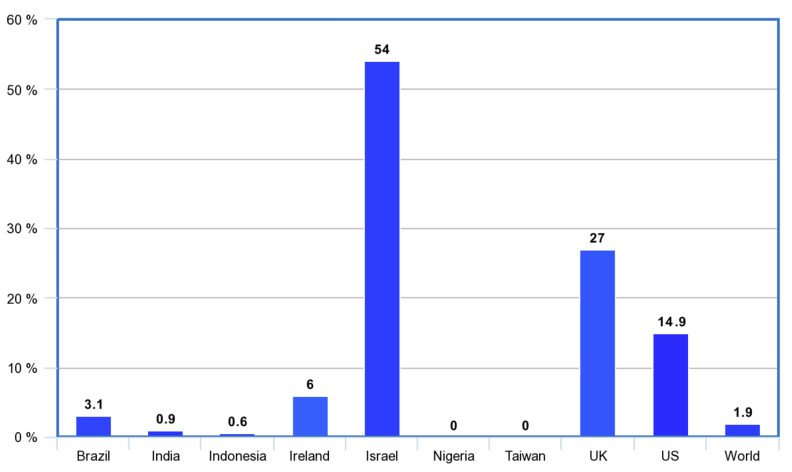
Percentage of population to receive at least one COVID-19 vaccine dose: 28 February 2021 [1].

Below are overviews of the strategies employed by nine regions in the launch of their COVID-19 vaccination drives, and how the processes unfolded in each. Percentages of populations to receive at least one dose by the end of February 2021 are included in the analysis, to give an idea of the effectiveness of the early stages of their immunization drives. Percentages of each population receiving their first dose by this date were: Brazil (3.1%), India (0.9%), Indonesia (0.6%), Ireland (6%), Israel (54%), Nigeria (0%), Taiwan (0%), United Kingdom (27%), United States (14.9%), World (1.9%) [1]. This is shown in Figure 1. Comparison also demonstrates the vaccine inequity that occurred globally. By the end of February 2021, close to 200 million shots had been given, covering approximately 1.9% of the global population, but 75% of these were in just 10 high-income countries (including the UK and US), while not a single dose had been administered in 130 low- and middle-countries with a combined population of 2.5 billion [2].

### 3.1. Brazil

South America’s largest country has a proud history of public health and vaccination success. The Brazilian National Immunization Programme was established in 1973, and since then incidence of vaccine-preventable illness has dramatically declined [3]. They are among the global leaders in offering free vaccines to their population, providing all 18 vaccines recommended by the World Health Organisation, and over 300 million jabs are administered in Brazil annually [4,5].

However, the Brazilian COVID-19 vaccine rollout was mired in controversy from its inception. The Ministry of Health’s national vaccination plan, published in December 2020, proposed 110 million shots to be given in the first half of 2021, enough for just under a quarter of the population of 211 million. Priority groups included medically vulnerable, health workers, elderly people and indigenous communities. The plan was rife with inconsistencies, did not include a designated start date and was met with overwhelming criticism and declared an inadequate response to the crisis [6].

President Bolsonaro consistently downplayed the severity of SARS-CoV-2, dismissing the infection as a simple flu and mocking social distancing measures. He declared that he would refuse to get vaccinated, and launched a bizarre rant suggesting that the Pfizer-BioNTech shot could cause women to grow beards, or turn vaccine recipients into crocodiles [7]. Despite purchasing millions of doses of the CoronaVac jab, he was vocally skeptical of its efficacy. A growing anti-vaccine movement in Brazil presented another challenge, highlighted by a resurgence in measles in 2018 after previous eradication of the infection [8]. A Datafolha poll in December 2020 indicated that 22% of Brazilians would refuse a COVID-19 vaccine, and 56% said they would not accept a Chinese vaccine [9].

Brazil’s health regulatory agency Anvisa approved two COVID-19 vaccines for emergency use in January; Britain’s Oxford–AstraZeneca and China’s CoronaVac. However, while other countries had been procuring jabs, the Brazilian government were hesitant, and by 18 January they had only secured 6 million doses. Immunization ambitions were further dented with publication of trial data from Brazil’s Butantan Institute indicating that the CoronaVac vaccine only provided 50.4% protection against COVID-19 infection, far lower than results with the same vaccine elsewhere [10]. Like in most places, supply has been a severe rate limiting factor in Brazil’s rollout. Contracts had reportedly been signed by mid-February for over 365 million doses of various vaccines, but still several cities like Rio de Janeiro were forced to delay scheduled inoculations due to massive shortages.

Distribution of vaccines to remote regions of Brazil is difficult both geographically and politically. There are at least 300 rural tribes in the country, comprising 900,000 people or 0.4% of the population, most of whom reside in the Amazon rainforest [11]. Access to some areas is by helicopter only, which is often viewed as an unwelcome intrusion. In February, a medical team arriving by air intending to vaccinate Jamamadi villagers on the São Francisco reservation in Amazonas, were met with fierce resistance and shot at with bows and arrows. “Religious fundamentalists and evangelical missionaries are preaching against the vaccine”, said Dinamam Tuxá, a leader of the Articulation of Indigenous Peoples of Brazil, which hinders efforts to set up vaccination drives in their communities [12]. The isolation of some Amazonian tribes can make it a near impossible feat even to communicate with members, let alone immunize them.

While public health officials desperately tried to get their struggling immunization drive on track, Brazil surpassed 10 million cases and 250,000 deaths in February [13,14]. The P1 strain, originating in the Amazonian city of Manaus, was becoming dominant in the country. This variant’s E484K mutation increased transmissibility and the virus’ ability to evade antibodies, threatening to reduce the protection provided by vaccination [15,16]. With a relentlessly rising death toll, health services on the verge of collapse, widespread anti-vaccination sentiment, an ambivalent leader and a haphazard immunization strategy, the odds continued to be heavily stacked against Brazil. By the end of February 2021, 8.4 million COVID-19 shots had been given in Brazil, with 3.1% of the population receiving at least one dose [1].

### 3.2. India

The Serum of Institute of India has the highest vaccine output of any pharmaceutical company in the world. They were producing 2.4 million COVID-19 vaccine doses daily by January 2021, and CEO Adar Poonawalla pledged to deliver 1 billion doses by the year’s end [17]. Two domestically produced vaccines, Covishield and Covaxin, received “restricted use approval in an emergency situation” by India’s national drug regulator CDSCO, allowing for their use prior to publication of complete clinical trial data [18]. Covishield is the Indian equivalent of the Oxford–AstraZeneca vaccine (AZD1222), manufactured by the Serum Institute. Covaxin was developed by Bharat Biotech and the National Institute of Virology [19]. Both vaccines have the advantage of low cost and simpler storage requirements (between 2 and 8 °C for up to 6 months) compared to the mRNA candidates.

Covaxin became a source of controversy because of its approval prior to completion of Phase 3 trials, with limited published data available on safety and efficacy [20]. Uncertainty surrounding this issue was reflected in its uptake. In the first week of the rollout, fewer than 30% of Delhi health workers in six government hospitals accepted vaccinations [21]. Three states (Chhattisgarh, Kerala and Punjab) announced in February they would not be administering any Covaxin doses until Phase 3 trial results were made available [22].

Communication from government officials on the topic was inconsistent. Covaxin was approved with the proviso that it would be administered in ‘clinical trial mode’. However, the distribution process had no plans for a control arm, or for monitoring the health of recipients [23]. A member of the National COVID-19 Task Force stated that the jab would be used “in an emergency situation” or “as a backup” to Covishield, but the head of the panel on vaccination strategy asserted 2 weeks later that “no vaccine is a backup to the other, both vaccines are equally important” [24,25]. Entering 2021, India had the advantage of a population generally supportive of vaccines. In November 2020, the Edelman Trust Barometer found 80% of Indians were willing to receive a COVID-19 vaccine, the highest rate of 28 countries surveyed [26]. However, the hasty approval of Covaxin without full access to data, combined with clumsy and contradictory messages from government, affected this trust and a subsequent survey in February found the figure to be just 42% [27].

The vaccination drive, which the government had been planning since August 2020, was launched on 16 January 2021. In preparation for the enormous scale of distribution, India also conducted three major nationwide ‘dry runs’ in the weeks leading up to the start date to prepare logistically and identify obstacles, covering areas in increasing size from 8 to 74 to 737 districts [28]. Vaccines would be given first to priority groups across 3006 vaccination hubs, with the number of centers gradually increasing throughout 2021 [29]. The priority population comprised approximately 30 million Indians, including front-line healthcare workers and over-50s, followed by people under 50 with underlying health conditions. The Indian government aimed at the start of 2021 to immunize 300 million people over the next 8 months [30].

Soon after the program commenced, India looked to vaccine exportation, donating millions of free jabs to low- and middle-income countries including neighboring Bangladesh, Bhutan and Nepal [31]. This became a contentious issue in India as only a small proportion of the country’s huge population had been vaccinated when exports began. The rationale given by Prime Minister Modi’s government was that supply exceeded capacity to vaccinate, but critics argued that this should lead to expansion of the rollout infrastructure, including mobilization of the private sector, rather than gifting extra doses internationally.

India’s generosity was applauded by many, and was in stark contrast to the vaccine nationalism seen in many high-income countries. However, with 1.366 billion people to immunize, the loss of these millions of doses was reflected in coverage in the early stages. By 28 February, as the national death toll exceeded 150,000, there had been 14.3 million inoculations in the country, meaning just 0.9% of Indians had received their first dose [1,32].

### 3.3. Indonesia

The Indonesian government secured deals in early 2021 for approximately 330 million vaccine doses from Sinovac, Oxford–AstraZeneca and Novavax, with those from Sinovac scheduled to arrive in the first quarter. President Joko Wikodo declared his intention to immunize two-thirds of the population of 270 million people over the next 15 months [33].

Indonesia was to deviate in COVID-19 vaccine rollout strategy in relation to group prioritization. While most nations commenced their campaigns by inoculating the elderly, Indonesia’s first groups were those of working age (18–59-year-olds), along with healthcare workers and public officials. There were three key reasons forming the basis for this decision, according to a COVID-19 vaccination spokesperson for Indonesia’s Ministry of Health. First, prioritizing this group would get people back to work sooner and stimulate the struggling economy. Second, as younger people were more likely to spread the infection to a higher degree due to greater levels of movement within communities, vaccinating them first should reduce transmission and indirectly protect the elderly and vulnerable. Third, the efficacy data in elderly patients for Sinovac’s CoronaVac jab were not as robust as for other approved COVID-19 vaccines [34,35].

Another unique element to Indonesia’s approach was the use of social media influencers to encourage vaccine confidence in the public. Following President Widodo, the second person in the country to be vaccinated was 33-year-old television personality Raffi Ahmad, and he posted online about the event to his 50 million Instagram followers. Various other musicians and celebrities were also included in the first round of inoculations [36].

A WHO and UNICEF survey in November 2020 found that 30% of Indonesians were reluctant to accept a COVID-19 vaccine, with concern about safety and efficacy the most common reasons given [37]. The vaccines were declared ‘halal’ (permissible under Islamic law) by the Indonesian Ulema Council in advance of the first doses being administered. This was a crucial declaration for the nation of 87% Muslims, as several vaccines in use today are ‘haram’ (forbidden) due to the use of pork-derived gelatin as a stabilizer [38]. The government declared that strict penalties would be handed out for those refusing to be vaccinated in South-East Asia’s hardest hit country. Proposed measures included fines, withdrawal of social aid and loss of access to administrative and public services [39].

Indonesia is an archipelago consisting of over 17,500 islands, around 6000 of which are inhabited, spanning 1.9 million squared kilometres. This would make the distribution and storage of vaccines highly challenging. Around 10,000 health centers throughout the country were allocated to receive shipments, some of these in remote settings without reliable electricity or cold storage, and power grids were under pressure to manage the demand for extra refrigerating units [33]. Indonesia has a steadily growing cold-chain industry, projected to become the world’s seventh largest by 2030. The government approached some of the cold-chain logistics companies, requesting them to divert all possible resources to facilitating vaccine rollout [40]. However, the number of logistical failures in storage and distribution remains high, “namely 10% and 20%, respectively”, according to the Indonesian Logistics and Forwarders Association [41]. Furthermore, the cold-chain industry is concentrated in Java in the country’s center, with maintenance facilities scarce in other regions.

By 28 February, over 36,000 COVID-related deaths had been registered in Indonesia [14]. At the same point, 2.69 million vaccine doses had been given, providing just 0.6% of the population with at least one dose [1].

### 3.4. Ireland

The 27 member states of the European Union agreed to approve and purchase COVID-19 vaccines as a bloc, and to distribute them among countries on a pro rata basis according to population size [42]. This decision was an act of European solidarity as well as a move to increase collective negotiating power with pharmaceutical companies. The European Medicines Agency (EMA) approved the Pfizer-BioNTech vaccine on 21 December 2020, and Ireland began inoculations the following week [43]. Two more vaccines, Moderna and Oxford–AstraZeneca, were soon added to the EMA approved list in 2021 and arrived in Ireland [44,45]. EU nations were frustrated throughout the early stages of the rollout with supply volumes lower than what had been promised. This was most apparent with the Oxford–AstraZeneca jab, with projected shipments around 60% lower than the agreed amount in the first quarter, leading to ferocious legal turmoil [46].

Top priority groups in Ireland were healthcare workers and the over-65s living in long-term care facilities [47]. By 21 January, over 120,000 doses had been administered, with around two thirds to healthcare workers and one third to nursing home residents and staff. The Irish government pledged that by September 2021, any adult resident who wanted one would be able to receive a COVID-19 vaccine [48]. The Irish government’s communication strategies were often criticized throughout the rollout. Priority lists, selection criteria and timelines shifted regularly. For example, the Oxford–AstraZeneca shot was widely proclaimed as a ‘gamechanger’ due to easier storage capabilities and endorsed by the Minister for Health as the leading vaccine for over-70s. However, the following week, Taoiseach Micheál Martin announced that Ireland would not be providing the jab to that cohort, citing a lack of reliable data supporting its efficacy in the elderly, following advice from the National Immunisation Advisory Committee [49].

Mistrust in the rollout was exacerbated by some high-profile scandals. In one incident, leftover doses in a Dublin hospital were given to relatives of staff members, who were not on the priority lists. These would have been discarded had they not been given that day, as they had already been drawn up from the vials, but nevertheless it caused outrage among the public [50]. Shortly afterwards, the Health Service Executive issued guidance on using surplus doses, whereby members from a standby list of frontline healthcare workers should be contacted in future similar events [51].

Locations for the vaccination of different groups were a cause for disagreement in Ireland. It was decided that GPs would immunize elderly citizens who were not living in nursing homes or long-term care. However, some GPs criticized the strategy, arguing that space was at a premium in their practices, especially with COVID-19 restrictions, and that it would take up valuable time and resources on an already heavily strained service. They advocated for relocating clinics to larger areas like churches or hotel function rooms that were left vacant due to the pandemic [52]. Forty dedicated mass vaccination centers were opened around the country, which would run for 12 h a day, 7 days a week [53].

Like many countries in the EU and globally, Ireland’s vaccine program started off slower than had been anticipated. From 15,000 doses administered in the first week of January, the number rose to 70,000 the week after, before falling to 40,000 in mid-February. Leaders expressed optimism however that supply and the speed of the operation was beginning to ramp up, forecasting 250,000 weekly inoculations by April. By 28 February, over 4300 COVID-related deaths had been recorded in Ireland and 439,000 vaccinations had been given, meaning that 6% of the population had received at least their first dose [1].

### 3.5. Israel

The Middle Eastern nation of 9 million stormed into the lead in the global COVID-19 vaccination race, inoculating 34% of citizens with at least one dose just four weeks into 2021. The next highest rate at the time was the United Kingdom on 11% [1]. Priority groups in Israel were the over-60s, health workers and clinically vulnerable individuals [54].

Their speedy rollout was the result of a deal struck early in the pandemic between Prime Minister Benjamin Netanyahu and Pfizer’s chief executive Albert Bourla. In exchange for a plentiful supply of doses, Israel would coordinate the world’s fastest vaccination drive and provide valuable safety and efficacy data to the pharmaceutical company [55]. Pfizer committed to provide 10 million doses for a cost of USD 245 million [56]. Israel also agreed a deal with Moderna worth USD 101 million. It was reported by the Israeli national broadcaster Kan that the government paid slightly over the global average for the vaccine doses, with a price of USD 23.50 per dose for both Pfizer and Moderna, compared to a cost of between USD 15–20 per dose for the mRNA vaccines in the EU and US [57].

The operation was scaled up rapidly, with daily doses increasing from 8000 on 20 December to 170,000 by late January [1]. Benefits of the program soon became apparent, as new cases reported in mid-February were at less than half the peak reached a month previously, with similar reductions in deaths. By the end of February, a total of approximately 5750 total COVID-related deaths had been recorded in Israel [58,59].

The arrangement with Pfizer also provided the pharmaceutical giant with an opportunity to measure the vaccine’s efficacy in a real-life setting. Results were excellent, matching the figures in clinical trials, which helped to further inspire confidence in other governments and populations in the utility of the jab [60]. Privacy concerns were raised however about the legitimacy of the data-sharing deal. Israel’s former deputy attorney general wrote “It is doubtful that the state has any authority at all to transfer the details of the vaccination recipient to any foreign body”. The government aimed to reassure critics that all data were anonymized and aggregated so that there could be no personal breaches of privacy [61].

With an abundant supply, challenges of overcoming vaccine hesitancy soon came to the fore in Israel. A survey in February on a representative sample of 503 Israelis found that of those who were unvaccinated at the time, a quarter did not intend to get immunized. Concern over side effects (41%) and efficacy (30%) were cited as the most common reservations [62]. The government implemented measures to strongly encourage uptake, mandating that only vaccinated individuals could avail of the newly reopened gyms, swimming pools, hotels and sporting events, providing evidence of immunity using a ‘green card’ system. The basis for this policy was supported by opinion surveys, as in the aforementioned vaccine-reluctant cohort, 31% responded that limitations on activities could persuade them to change their minds [63].

Israel’s unparalleled success in the early phase was largely due to the Pfizer deal, but also thanks to an immense logistical effort. Firstly, all Israeli citizens must be registered to one of the country’s four Health Management Organizations, which retain comprehensive electronic health records on all members, and have strong capabilities for electronic communications with them [64]. This has been useful both for sharing data with Pfizer and also in coordinating vaccine distribution in the country, for example in identifying suitable vaccine recipients and informing them of their appointments via call centers, apps and websites [65]. Furthermore, given their geopolitical situation, the government has invested over the years in preparing for large-scale emergencies in a so-called ‘all hazards’ approach [66]. They periodically carry out drills in response to a variety of logistical challenges, coordinated by the HMOs. Previous scenarios, in a time before COVID-19, involved planning for mass vaccination campaigns, such as in response to military bioterror attacks [67]. By the end of February, Israel had inoculated 54% of the population with at least one dose, administering a total of 8.11 million jabs, and becoming the first country globally to provide some level of immunological protection against SARS-CoV-2 to over half of citizens [1].

### 3.6. Nigeria

African nations have been marginalized in efforts to vaccinate against COVID-19, as lack of purchasing power prevented them from brokering deals with pharmaceutical companies, while wealthy nations secured and hoarded hundreds of millions of doses. The continent of 1.1 billion people would be almost entirely reliant on the African Union and COVAX to obtain doses for their immunization campaigns [68].

Despite rhetoric from world leaders in 2020 emphasizing the need for equitable global access to COVID-19 treatments and technologies, the reality that unfolded as the rollout commenced was severely imbalanced. Rich countries quickly snapped up most available vaccines and by September 2020, nations comprising 13% of the world’s population had secured 51% of the future supply of available doses [69]. If the trend continues unabated, the pandemic will continue to rage in low- and middle-income countries, with deleterious effects on the overall health, safety and economy of the world. As WHO Director-General Dr. Tedros Ghebreyesus warned, “Let me be clear, vaccine nationalism will prolong the pandemic, not shorten it”, describing the global vaccine rollout as a “catastrophic moral failure” [70,71].

Glaring inequalities notwithstanding, Dr. Faisal Shuaib, head of Nigeria’s National Primary Health Care Development Agency, published a plan to immunize 40% of the population by the end of 2021, and a further 30% in 2022. For a nation of 200 million, this amounted to 280 million doses, assuming two-shot regimens. Priority groups were frontline healthcare workers, first responders, the elderly, medically vulnerable and national leaders [72]. Nigeria were allocated 16 million doses of Oxford–AstraZeneca by the COVAX global vaccine-sharing scheme for the first quarter of 2021, backed by the WHO, CEPI and Gavi, with the first shipment scheduled to arrive in March [73]. Prior to this arrival, by 28 February, Nigeria reported a total of 1915 deaths from COVID-19 [14,74].

Regarding hesitancy, 65% of a representative group in Africa’s most populous country surveyed in October 2020 said they would accept a COVID-19 vaccine [75]. However, Nigerians can recall in 2003 a vaccine boycott fueled by public mistrust, that had damaging consequences. The Global Polio Eradication Initiative aimed to immunize 15 million children in West and Central Africa. At the time, 45% of global polio cases were in Nigeria [76]. Religious and political leaders in three states asserted that these vaccines could cause HIV, cancer and infertility, and campaigns collapsed in these regions. Cases rose by 30%, and new vaccine-resistant strains developed. Polio was finally eradicated from Africa in 2020, after 9 billion oral doses and 24 years, with Nigeria being the last country on the continent to be declared polio-free [77,78]. Lessons can be learned from this boycott, as the WHO, COVAX and governments endeavor to prevent the spread of misinformation and ensure that trust in COVID-19 vaccines is maintained.

The mRNA vaccines must be stored at colder temperatures than most vaccines in use today, and Nigeria acquired three ultra-cold freezers in January 2021, anticipating a shipment of Pfizer-BioNTech doses from COVAX [79]. The delivery was subsequently cancelled, and Nigeria was allocated Oxford–AstraZeneca jabs instead. The news was welcomed by Dr. Shuaib who remarked that “Our plan is not to over invest in ultra-cold chain equipment given that there are other vaccines that can be kept between 2–8 °C” [73]. Low- and middle-income nations may prefer these vaccines, sacrificing some efficacy for lower costs and less intensive storage requirements. Over 99% of the doses in COVAX’s first delivery were Oxford–AstraZeneca and Covishield, which can be stored at refrigerator temperature for months and cost USD 2–3 per dose (compared to USD 15–20 for Pfizer or Moderna) [8,73].

Without the interventions of COVAX, many poorer nations like Nigeria would likely be forced to wait until the global North had satisfied their demand for vaccines, before receiving any shipments. The organization’s aim is to procure and distribute 2 billion vaccine doses in 2021, including at least 1.3 billion doses to the poorest 92 countries, enough to immunize a quarter of their citizens [80]. When we look back at the COVID-19 pandemic and its horrific impact, the COVAX initiative may well be remembered as the most important thing we as a global community did to protect ourselves and each other from this devastating virus.

### 3.7. Taiwan

Taiwan performed exceedingly well at COVID-19 suppression even before the introduction of vaccines. They had recorded a total of just 942 cases and nine deaths since the start of the pandemic as of 28 February 2021 [14,81]. Contact tracing, testing and quarantine protocols were all in place by the time their first COVID-19 case was detected on 21 January, and the East Asian region avoided economically damaging lockdowns while protecting the health of their people [82]. Experience from the SARS epidemic in 2003 (when they recorded 37 deaths), meant the government and citizens were more aware of the risks and the necessary preventive measures for a contagious respiratory coronavirus, compared to most countries [83]. They were also the first to inform the WHO of the potential for human-to-human transmission of this novel pathogen [84].

The Taiwanese established the Electronic Fences System (IEFS), a collaboration between the Central Epidemic Command Center (CECC) and mobile phone networks used to detect quarantine rulebreakers and to identify potential close contacts with GPS technology [85]. The government also focused on engendering trust and cooperation in the public as a key part of their plan, compensating individuals in quarantine with USD 33 per day, sending masks and information to every household, and providing free online access to movies and exercise videos, to improve the experience of being forced to stay at home [86].

Low case and death numbers meant the provision of vaccines was not as urgent in Taiwan as elsewhere, but immunization would always be necessary to return to normality. The CECC confirmed purchase of 20 million doses of COVID-19 vaccines in January. Half of these were from Oxford–AstraZeneca, a quarter were through the COVAX initiative, and a quarter from an undisclosed source [87]. Their stated objective was to reach herd immunity by inoculating 65% of the population with around 30 million doses [88]. As well as sourcing shots internationally, three developers in Taiwan had vaccine candidates in clinical trials, namely Adimmune Corporation, Medigen Vaccine Biologics and United Biomedical [89]. The CECC also built an IT system for keeping track of recipients and reporting any adverse events [90].

Taiwan’s priority list was unique in that only the occupation was considered, not age or health status. The first priority group was 332,000 healthcare workers, followed by 140,000 frontline epidemic prevention personnel, borough chiefs, and drivers who transport potentially infected passengers from the airport to quarantine facilities. The third group comprised 90,000 police and military and officers. Next were 158,000 who maintain the social welfare system, and around 200,000 soldiers [91,92]. By late February 2021, Taiwan was yet to immunize any citizens, but 200,000 vaccine doses had been allocated to the region in the first round of distribution from COVAX, scheduled to arrive in early March [93,94,95].

### 3.8. United Kingdom

The UK became the first country to administer a COVID-19 vaccine outside of a clinical trial on 8 December 2020 [96]. Britons may point to this quick start while the European Medicines Agency spent extra weeks deliberating, as an example of the kind of efficiency that can be achieved as a now former member of the European Union.

The British government invested heavily in immunization throughout this crisis. On top of GBP 280 billion spent combatting the pandemic, they allocated GBP 11.7 billion to the research, development and distribution of COVID-19 vaccines [97]. They had procured 367 million doses by January 2021 from seven different companies, for their population of just 66.5 million people [98]. They also took on significant manufacturing commitment for their supply, producing the American Novavax vaccine in England’s North-West, French company Valneva’s jab in Scotland, and the domestically developed Oxford–AstraZeneca jab at two locations in Oxford and Keele [99,100].

Oxford University’s Jenner Institute had been preparing for ‘Disease X’, a new and unknown pathogen, since before the arrival of COVID-19. They created the ChAdOx1 vaccine using a chimpanzee common cold adenovirus as a viral vector. which could be genetically altered for different diseases, and had already tested it with several viruses by the time Chinese scientists published the full genetic code of SARS-CoV-2 on 11 January 2021 [101]. The ChAdOx1 nCoV-19 (or AZD1222) jab delivers instructions to cells to produce the spike protein, and the immune system develops antibodies against it [102]. An interim analysis in December 2020 found the overall efficacy was 70.4%, based on data from 11,636 participants [103,104]. The vaccine had been approved in 43 countries by early February 2021 [105]. The UK was spared the supply disputes that the EU and Oxford–AstraZeneca faced, as the British government had pre-emptively signed deals with the manufacturer for 100 million doses back in June 2020 [106,107,108].

The national COVID-19 vaccination plan was published online, outlining the program clearly under four headings: supply, prioritization, places and people. Information also covered costs and quantities of different vaccines, ongoing research into efficacy against mutant strains, and many other topics [98]. The website updated daily with numbers receiving first or second doses, stratified into each of the UK’s four nations. The clarity of communication was impressive, in an area where many other governments have been found wanting.

The rollout was coordinated centrally by the National Health Service, across over 1600 sites including GP practices, pharmacies, hospitals and mass vaccination sites [109]. This meant 97% of the population would live within 10 miles of a vaccination site [110]. Top priority groups comprised care home residents and care home workers, frontline health and social care workers, over-75s and medically vulnerable people over 70, amounting to a total of 15 million people [111]. These four groups accounted for over 90% of the UK’s COVID-19 related deaths in 2020, and the government’s aim was to immunize these cohorts by mid-February, a target that would require just under 400,000 jabs a day [112].

By the end of February 2021, the UK had recorded the fifth highest number of fatalities from COVID-19 in the world and the highest in Europe, with over 123,000 deaths [14]. However, at the same point, over 21 million vaccinations had been administered and 27% of Brits had received at least one dose, ranking them third in the world behind Israel and UAE, with the rollout moving onto its second phase and new daily cases and deaths steadily declining [1,113]. Buoyed by the success of the immunization drive thus far, Prime Minister Boris Johnson outlined an optimistic four-step plan to remove “all legal limits on social contact” by the summer [114].

### 3.9. United States

The initial response of the US government under President Trump was to dismiss the severity of the virus and within weeks, they had reached the world’s highest death toll [115]. However, the Americans embraced the challenge of vaccine development, and made huge investments that paid dividends for themselves and many other countries globally.

Boston-based Moderna Therapeutics was the first pharmaceutical company to begin testing a COVID-19 vaccine candidate on human volunteers [116]. They received nearly USD 1 billion in mRNA research funding from the government’s Biomedical Advanced Research and Development Authority in the first half of 2021 [117]. The candidate, mRNA-1273, encodes the SARS-CoV-2 spike protein so that immune cells synthesize the protein and then develop antibodies against it [118,119]. A Phase 3 trial with 30,420 participants demonstrated 94.1% efficacy at preventing COVID-19 infection. The findings were also evident in the over-65s age group [120]. The US Food and Drug Authority authorized Moderna’s vaccine for emergency use in December 2020, as well as the Pfizer-BioNTech jab [121,122]. They would go on to secure deals by early February for 300 million doses of each of the two mRNA vaccines [123,124].

The rollout was widely criticized for a chaotic opening and lack of national leadership under the outgoing Trump administration. By mid-January, 31 million doses had arrived, but only 12 million had been administered [125]. Congress approved USD 3 billion in funding in December to facilitate vaccine distribution, but that money had not arrived in states by the time of Trump’s departure [126].

The Centres for Disease Control and Prevention made recommendations for group prioritization in the vaccine rollout [127]. Phase 1a included healthcare staff and long-term care facility residents; phase 1b was frontline essential workers, and over-75s; and phase 1c was over-65s, people with underlying conditions and other essential workers [128,129]. However, each of the 50 states had autonomy to design their own ranking system. They mostly complied with phase 1a guidelines but many states chose to move young and healthy essential workers down the list in favor of elderly citizens who were more vulnerable to becoming severely ill with the infection [130].

Variations in strategies may have contributed to a lack of clarity from the public’s perspective. A survey by the Kaiser Family Foundation in January found that 60% of respondents did not have enough information about when or where they would receive the vaccine, and two-thirds felt that the federal and state governments were doing a ‘poor’ or ‘fair’ job in rolling out the vaccines. Less than half of Black adults interviewed believed that distribution efforts were taking into account the needs of Black people [131]. The survey found that 46% of Americans were ‘very likely’ to agree to receive the vaccine if offered, with reluctance highest in African-American and Hispanic groups [131]. The CDC reported in February that across 11,460 COVID-19 nurse-led vaccination clinics, only 37.5% of staff had opted to be immunized. Frequently cited reasons for hesitancy included ‘perceived rapidity of vaccine development, and inadequate information about vaccine safety and side effects’ [132].

On 24 February 2021, the FDA concluded that Johnson & Johnson’s single-shot COVID-19 vaccine was safe and effective, paving the way for its approval, which would make it the third vaccine available to the nation of 328 million people [133]. A projected 20 million doses could be delivered in March, 1 year after the federal government invested USD 456 million in its development [134]. This positive news contrasted with another horrific milestone in the country as the number of Americans who had died with the virus exceeded 500,000 [14].

When taking office in January 2021, President Biden pledged to immunize 100 million Americans in his first 100 days [135]. He swiftly re-joined the World Health Organisation, emphasized the importance of masks and social distancing measures to all citizens, and committed to the COVAX initiative [136]. By 28 February, with over 75 million shots given, 14.9% of US citizens had received at least one dose of a COVID-19 vaccine [1,8,113].

## 4. Discussion

The early phase of these nine COVID-19 vaccine rollouts varied significantly in strategies for preparation and execution. If the measurable outcome here is the proportion of the population who were inoculated 2 months into 2021, results also covered a wide spectrum. We can reflect on three key aspects of the immunization programs that influenced success: procurement, communication and distribution. The percentages of each population receiving their first dose by 28 February 2021 were: Brazil (3.1%), India (0.9%), Indonesia (0.6%), Ireland (6%), Israel (54%), Nigeria (0%), Taiwan (0%), United Kingdom (27%), United States (14.9%), World (1.9%).

### 4.1. Procurement

Before the rollout can begin, vaccine doses must be acquired. The UK and US invested heavily and early in the pandemic to create homegrown jabs. They also hedged their bets, as the UK brokered deals with seven pharmaceutical companies, and the US supported several candidates, not all of which would complete trials. This investment and preparation ensured that once vaccines were approved for use, these two nations had sufficient supply to quickly begin immunizing large numbers of citizens. India developed jabs domestically too and had the infrastructure to produce doses at an astonishing speed. However, with the world’s second largest population, and a controversial decision to export vaccines internationally, their coverage rate at this point was far lower than their British and American counterparts. The other six regions had no COVID-19 vaccine production of their own at this time and subsequently relied on importation. Significant variation was evident in how this worked out for each. Brazil’s inefficiency and skepticism from leaders slowed down their initial supply greatly. They signed contracts for hundreds of millions of doses, but inadequate planning and poor governance in a country with historical vaccination success led to them falling short of their potential in the early stages. Indonesia also managed to secure hundreds of millions of doses at the start of 2021, but most of these would not arrive for several months. This hindered the rollout and was a major contributing factor to a devastating wave of infections in the first half of the year. Brazil and Indonesia both procured a majority of their vaccines from Sinovac, for which clinical trials demonstrated inferior results compared to some of the other candidates. Ireland’s procurement was in line with the rest of the EU bloc, which eliminated the need to negotiate with suppliers directly. Several deals were made with Pfizer-BioNTech, Moderna and Oxford–AstraZeneca, but a promising start was dented as ambitious contractual obligations were not met by suppliers, leading to furious legal battles and a slower than anticipated rollout. Similar to Indonesia, Taiwan secured enough doses for a large cohort of their population but would have to wait some months before receiving any of them, again due to manufacturers making deals with countries that they simply could not meet in the urgent timeframe. Nigeria, like most of Africa, lacked the financial capability to purchase anywhere near enough doses directly, and awaited their first COVAX shipments, scheduled to arrive in March 2021, before they could begin their immunization drive in earnest. Of the nine case studies, it was Israel’s negotiation with Pfizer that proved a masterstroke in granting them quick access to millions of doses in the first months of the rollout and allowing them to rapidly scale up their campaign, to the amazement and envy of the rest of the world.

### 4.2. Communication

An immunization drive will not be successful without adequate communication strategies. In an era of widespread anti-vax sentiment and disinformation, the importance of this step in the process is paramount. India’s rushed approval of the Covaxin vaccine without completion of phase 3 trials proved to harm the public’s confidence in the program and led to a drop in uptake of the jab. Brazil’s President Bolsonaro would actively discourage his citizens from following public health measures throughout the pandemic, and made exaggerated false claims about vaccine side effects, again reducing public buy-in to the rollout. In the United States, President Trump mirrored his South American counterpart in dismissing the severity of COVID-19 illness, despite hundreds of thousands of deaths in the country, and even after he himself became sick with the virus. This rhetoric would serve to undermine efforts to encourage Americans to get the jab, until a changing of the guard in January 2021 shifted the narrative towards the importance of following public health advice. Ireland’s communication strategies could be inconsistent at times, and policy changes caused confusion and skepticism, particularly in relation to the Oxford–AstraZeneca jab, but they had no major setbacks or any deliberately misleading messages such as those in Brazil and the US. The UK government were also guilty at times of underestimating the virus, but once the rollout commenced, they performed well at communicating with the British public in relation to vaccines. Online published information about the rollout was clear, accessible, relevant and encouraged uptake from its citizens. Israel’s major success in the first few months of 2021 would be threatened by allegations of breaching citizens’ privacy rights by providing health data to Pfizer. A communication challenge for them would be to convince their citizens that confidentiality would be protected, and that early access to so many vaccines was worth the trade-off. Indonesia employed a unique approach to addressing vaccine hesitancy, by immunizing social media influencers before any other members of society, in an effort to engender support in the population. This policy was not replicated in any of the other eight rollouts, as they instead would prioritize elderly and vulnerable cohorts in the first stages. At the time of analysis, Taiwan and Nigeria had not begun their rollouts, but communication lessons can be learned from the two case studies. In relation to quarantining, the Taiwanese government focused on encouraging cooperation from citizens, by financially rewarding them for complying with regulations. Additionally, Nigeria will be acutely aware of the ability of religious or political leaders to sway opinion against immunization, based on a past experience almost two decades previously when a polio vaccination campaign was derailed by propaganda and misinformation.

### 4.3. Distribution

The most important aspect of the drive is the logistics behind physically getting vaccines into people’s arms. Following on from differences in procurement and communication, these nine regions also demonstrated varying degrees of success in this crucial area. In the United States, a lack of coordination between federal and state government and discrepancies between states on prioritization policy hindered the rollout initially before it started to gather pace. The approval of the one-shot Johnson & Johnson vaccine facilitated a welcome increase in speed. Taiwan and Nigeria would not begin the distribution aspect of their campaigns until the first COVAX deliveries arrived in March 2021, after the period covered by this analysis. Geographical landscapes were an important factor to consider in many countries including Brazil and Indonesia. The South Americans faced a complex challenge which would not figure into most nations’ plans, in aiming to immunize uncontacted tribes of the Amazon rainforest, in some cases being met with violent retaliation. Indonesia’s thousands of islands complicated their vaccine distribution significantly, especially in relation to maintaining the cold chain across millions of squared kilometers. In contrast to those two, the UK, with a far more navigable geography, could set up hundreds of vaccination sites nationwide so that citizens may be as close as possible to a center, allowing most people to get vaccinated a reasonably short distance away. Israel received plaudits for their canny bargaining with Pfizer, but also used a comprehensive registry of population health data and pre-pandemic mock vaccination drills to their benefit. The IT systems and the logistical training allowed the Israelis to be prepared for a highly efficient distribution process once the precious jabs arrived on their soil. Ireland utilized hospitals, GPs and dedicated vaccination centers for their rollout, with disagreements regarding the optimal locations to use, but overall managed to slowly but steadily increase the weekly number of jabs. India planned for months in advance of what would be a monumental effort to immunize well over one billion people. The logistical challenges to cover a population of this magnitude, particularly in a lower-middle income country, are immense, and they performed multiple dry runs of the distribution process in advance of the eventual launch.

## 5. Conclusions

These nine case studies have outlined some features that have been effective in launching national vaccination campaigns against COVID-19. Essential ingredients for success include good governance, early support and investment in vaccine development and manufacturing, ensuring robust health and information technology systems to handle the logistical challenges of the operation, and clear and consistent communication strategies to maintain the trust of citizens. The global community can look at these examples and others to ensure we are maximizing our potential in the ongoing efforts to vaccinate against this virus. When the time comes to face the next pandemic, we can also learn from our experiences with COVID-19 to be better prepared to rapidly immunize the world against a novel micro-organism.

To build on the findings from this research, it would be useful to conduct further analysis of the steps taken by these nine regions as this pandemic has evolved, and to evaluate how governments have reacted to new challenges, such as the mutation of COVID-19 and the threat posed by variants, the controversies surrounding the need for booster doses, and decisions about lifting lockdown restrictions once certain vaccination targets have been met. A follow-up graph reflecting proportions of the nine populations vaccinated by 28 August 2021 is shown in Appendix A. Moreover, the analysis could be expanded by looking at more countries outside of the nine case studies covered in this article, to further add to our understanding of the pros and cons of various rollout strategies.

The figures presented in this article highlight the gross inequity that has plagued the launch of the global COVID-19 vaccine rollout. There is a correlation visible in the data here between national wealth and the success of the early stages of immunization program. High-income countries like Israel, UK and US were able to rapidly accelerate their rollouts and immunize large numbers of their populations in early 2021, while poorer countries such as Nigeria and Indonesia lagged behind. Many low- and middle-income countries relied on shipments from COVAX and could not broker deals with pharmaceutical companies to secure doses, with the same speed and volume as their high-income counterparts, if at all.

A joint statement by UNICEF Executive Director Henrietta Fore and WHO Director-General Dr. Tedros Ghebreyesus in February 2021 criticized this lack of equity and emphasized the importance of global collaboration. They described vaccine nationalism as a “self-defeating strategy will cost lives and livelihoods, give the virus further opportunity to mutate and evade vaccines and will undermine a global economic recovery” and called on leaders to “look beyond their borders and employ a vaccine strategy that can actually end the pandemic and limit variants.” If governments continue to look inward and protect only their own citizens at the expense of the global population, this will inevitably lengthen the duration and exacerbate the severity of this pandemic, also increasing the likelihood of more virulent and vaccine-resistant COVID-19 strains arising. To conclude, they reaffirmed to the world that the only way to emerge from this crisis is through international cooperation. “COVID-19 has shown that our fates are inextricably linked. Whether we win or lose, we will do so together” [137].

## Figures and Tables

**Figure A1:**
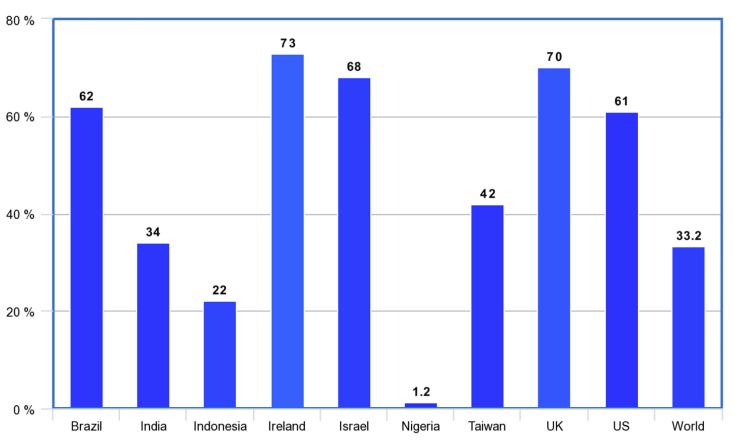
Percentage of population to receive at least one COVID-19 vaccine dose: 28 August 2021 [1].

## Data Availability

Publicly available datasets were analyzed in this study. This data can be found here: https://ourworldindata.org/covid-vaccinations, accessed on 9 January 2021.

## Appendix A

Updated figures as of 28 August 2021. By that date, 33.2% of the world’s population had been partially or fully immunized against COVID-19, but only 1.6% of people in low-income countries had received at least one dose [1].

## References

[1] Ritchie H., Mathieu E., Rodés-Guirao L., Appel C., Giattino C., Ortiz-Ospina E., Hasell J., Macdonald B., Beltekian D., Roser M. (2021). Coronavirus Pandemic (COVID-19). Our World in Data. https://ourworldindata.org/covid-vaccinations..

[2] Yamey G. (2021). Rich countries should tithe their vaccines. Nature.

[3] Domingues C.M.A.S., da Silva Teixeira A.M., Carvalho S.M.D. (2012). National immunization program: Vaccination, compliance and pharmacovigilance. Rev. Inst. Med. Trop. São Paulo.

[4] Ghc.fiu.edu. https://ghc.fiu.edu/_assets/docs/immunization-lais-martins.pdf..

[5] Financial Times. Brazil’s Vaccination Hindered by Bottlenecks and a Sceptical Leader. https://www.ft.com/content/a35515fd-4c73-4f1e-9e90-1ec3dd39deda.

[6] The Guardian. “We’re Being Left Behind”: Anger and Confusion in Brazil as Vaccine Program Lags. https://www.theguardian.com/world/2020/dec/31/brazil-coronavirus-vaccine-jair-bolsonaro.

[7] The Independent. Bolosonaro says Covid Vaccine May Turn People into Crocodiles in Bizarre Rant. https://www.independent.co.uk/news/world/politics/bolosonaro-covid-vaccine-brazil-crocodiles-b1776753.html.

[8] Castro M. Anti-Vax Movement Growing in Brazil as Measles Makes a Comeback. The Brazilian Report. https://brazilian.report/power/2019/08/05/anti-vax-movement-brazil-measles-comeback.

[9] Reuters Vaccine Refusal in Brazil Grows to 22%, Most Reject Chinese Shot: Poll. https://www.reuters.com/article/us-health-coronavirus-brazil-idUSKBN28M0VC.

[10] Krammer F. (2020). SARS-CoV-2 vaccines in development. Nature.

[11] Brazilian Indians. Survivalinternational.org. https://www.survivalinternational.org/tribes/brazilian.

[12] The Guardian. Brazil: Missionaries ‘Turning Tribes Against Coronavirus Vaccine’. https://www.theguardian.com/world/2021/feb/11/brazil-missionaries-turning-tribes-against-coronavirus-vaccine.

[13] Worldometers. Brazil COVID—Worldometer. https://www.worldometers.info/coronavirus/country/brazil/.

[14] Worldometers. *COVID*—*Worldometer*. https://www.worldometers.info/coronavirus.

[15] BBC. What Are the Delta, Gamma, Beta and Alpha Covid variants?. https://www.bbc.com/news/health-55659820.

[16] Jangra S., Ye C., Rathnasinghe R., Stadlbauer D., Krammer F., Simon V., Martinez-Sobrido L., García-Sastre A., Schotsaert M., Personalized Virology Initiative Study Group (2021). SARS-CoV-2 spike E484K mutation reduces antibody neutralisation. Lancet Microbe.

[17] Serum Institute of India. https://www.seruminstitute.com.

[18] Kapur M. India Approved its Own Covid-19 Vaccine before it Completed its Human Trials. Quartz. https://qz.com/india/1951939/how-india-approved-bharat-biotechs-covaxin-vs-covishield.

[19] Bharat Biotech. https://bharatbiotech.com/covaxin.html.

[20] ClinicalTrials.gov. Whole-Virion Inactivated SARS-CoV-2 Vaccine (BBV152) for COVID-19 in Healthy Volunteers. https://clinicaltrials.gov/ct2/show/NCT04471519.

[21] Thiagarajan K. (2021). Covid-19: India is at centre of global vaccine manufacturing, but opacity threatens public trust. BMJ.

[22] Sharma N. Covaxin in Punjab and Kerala Freezers till Phase-3 Trial Results. Economic Times. https://economictimes.indiatimes.com/news/politics-and-nation/covaxin-in-punjab-and-kerala-freezers-till-phase-3-trial-results/articleshow/80933113.cms.

[23] Mohapatra P.R., Mishra B. (2021). Regulatory approval of COVID-19 vaccine for restricted use in clinical trial mode. Lancet Infect. Dis..

[24] Hindustan Times. Bharat Biotech’s Covaxin can be used as backup, says AIIMS Director Dr Randeep Guleria. https://www.hindustantimes.com/india-news/great-day-for-the-country-says-aiims-director-after-2-covid-19-vaccines-get-govt-nod/story-Q5KUMGZEKnakU5Cnm6GMCK.html12.

[25] Arora K. India Will Treat Both Vaccines ‘Equally’, Recipients Won’t Get a Choice: V.K. Paul. Science—The Wire. https://science.thewire.in/health/india-covaxin-covishield-equally-vk-paul-choice.

[26] Edelman Trust Barometer. https://www.edelman.com/trust/2021-trust-barometer.

[27] India vs. Disinformation. 42% Indians Now Willing to Take Covid-19 Vaccine: Survey. https://www.indiavsdisinformation.com/20210204/42-indians-now-willing-to-take-covid-19-vaccine-survey.

[28] BBC. Coronavirus: How India Is Readying for its Massive Vaccine Drive. https://www.bbc.com/news/world-asia-india-55467980.

[29] Krishna N., Das M., Reuters ‘Go For It,’ Says First Person Vaccinated in India’s Massive COVID-19 Campaign. https://www.reuters.com/article/us-health-coronavirus-india-vaccine-idUSKBN29L04M.

[30] Shyam A., Krishna S. COVID-19: How India Will Vaccinate 300 Million People in 8 Months. Gulf News. https://gulfnews.com/special-reports/covid-19-how-india-will-vaccinate-300-million-people-in-8-months-1.1611039261355.

[31] NDTV Why India Is Exporting Vaccines But Not Selling Them For Public Use. https://www.ndtv.com/india-news/coronavirus-vaccine-why-indias-vaccines-are-exported-but-not-sold-in-market-for-public-use-2359713.

[32] Worldometers. India COVID—Worldometer. https://www.worldometers.info/coronavirus/country/india/.

[33] The Jakarta Post. ‘Disaster’: Indonesia’s Vaccine Campaign Lags Rampaging Pandemic. https://www.thejakartapost.com/news/2021/02/05/disaster-indonesias-vaccine-campaign-lags-rampaging-pandemic.html.

[34] BBC. Indonesia Coronavirus: The Vaccination Drive Targeting Younger People. https://www.bbc.com/news/world-asia-55620356.

[35] ClinicalTrials.gov. Efficacy, Safety and Immunogenicity Study of SARS-CoV-2 Inactivated Vaccine. https://www.clinicaltrials.gov/ct2/show/NCT04508075.

[36] Lamb K., Reuters Instagram influencers are a vaccine priority in wary Indonesia. https://www.reuters.com/article/us-health-coronavirus-indonesia-influenc-idUSKBN29J14E.

[37] COVID-19 Vaccine Acceptance Survey in Indonesia. https://covid19.go.id/storage/app/media/Hasil%20Kajian/2020/November/vaccine-acceptance-survey-en-12-11-2020final.pdf.

[38] Reuters Indonesian Clerics Declare Sinovac’s COVID-19 Vaccine Halal. https://www.reuters.com/article/health-coronavirus-indonesia-vaccine-idINKBN29D1DO.

[39] Arab News. Indonesia Gets Tough on COVID-19 Vaccine Skeptics as Phase Two of Inoculations Begins. https://arabnews.com/node/1810636/world.

[40] Hutton J. Indonesia’s vaccine roll-out comes up against cold reality of limited logistics. The Straits Times. https://www.straitstimes.com/asia/se-asia/indonesias-vaccine-rollout-comes-up-against-cold-reality-of-limited-logistics.

[41] Translog. Indonesian Cold Chain Market Grows Fast, Here’s the Logistics Player’s Point of View. https://translogtoday.com/2020/09/19/indonesian-cold-chain-market-grows-fast-heres-the-logistics-players-point-of-view.

[42] European Movement Ireland. https://www.europeanmovement.ie.

[43] European Medicines Agency EMA Recommends First COVID-19 Vaccine for Authorisation in the EU. https://www.ema.europa.eu/en/news/ema-recommends-first-covid-19-vaccine-authorisation-eu.

[44] European Medicines Agency Spikewax (Previously COVID-19 Vaccine Moderna). https://ema.europa.eu/en/medicines/human/EPAR/covid-19-vaccine-moderna.

[45] European Medicines Agency EMA Receives Application for Conditional Marketing Authorisation of COVID-19 Vaccine AstraZeneca. https://www.ema.europa.eu/en/news/ema-receives-application-conditional-marketing-authorisation-covid-19-vaccine-astrazeneca.

[46] Guarascio F., Siebold S., Reuters EU Locks Horns with AstraZeneca on Vaccine Deliveries Amid ‘Supply Shock’. https://www.reuters.com/business/healthcare-pharmaceuticals/eu-locks-horns-with-astrazeneca-vaccine-deliveries-amid-supply-shock-2021-01-25.

[47] Health Service Executive. https://www2.hse.ie/screening-and-vaccinations/covid-19-vaccine/rollout-covid-19-vaccines-ireland.html.

[48] Irish Times. Every Adult in Ireland to Have Covid-19 Vaccine by September, Minister for Health Says. https://www.irishtimes.com/news/politics/every-adult-in-ireland-to-have-covid-19-vaccine-by-september-minister-for-health-says-1.4464245.

[49] Neville E. Over 70s will not be given ‘game-changer’ AstraZeneca Covid-19 vaccine—Taoiseach. Irish Examiner. https://www.irishexaminer.com/news/politics/arid-40220000.html.

[50] Irish Times. Coombe Begins Investigation into Vaccination of Family Members of Staff. https://www.irishtimes.com/news/health/coombe-begins-investigation-into-vaccination-of-family-members-of-staff-1.4463568.

[51] Health Service Executive. https://www.hse.ie/eng/services/news/newsfeatures/covid19-updates/covid-19-vaccine-materials/sequencing-of-covid-19-vaccination-of-frontline-healthcare-workers.pdf.

[52] Irish Times. Doctors Differ over Rollout of Covid-19 Vaccine to Over-85s in GP Clinics. https://www.irishtimes.com/news/health/doctors-differ-over-rollout-of-covid-19-vaccine-to-over-85s-in-gp-clinics-1.4485783.

[53] RTE News. People Will Begin to See Light Emerge as Vaccinations Increase. ‘Light Will Emerge’ as Vaccinations Rise—HSE Chief. https://www.rte.ie/news/2021/0222/1198546-coronavirus-ireland.

[54] BBC. Coronavirus: Israel Leads Vaccine Race with 12% Given Jab. https://www.bbc.com/news/world-55514243.

[55] Financial Times. How Israel Secured More Vaccines than it Can Use. https://www.ft.com/content/3aae4345-46cc-4636-a3f9-a93a6762f87f.

[56] Politico The Secrets to Israel’s Coronavirus Vaccination Success. https://www.politico.eu/article/israel-coronavirus-vaccine-success-secret.

[57] Times of Israel. Israel Said to be Paying Average of $47 Per Person for Pfizer, Moderna Vaccines. https://www.timesofisrael.com/israel-said-to-be-paying-average-of-47-per-person-for-pfizer-moderna-vaccines.

[58] Vox. Israel Outpaced the World in Vaccinations. Now it’s Seeing the Results. https://www.vox.com/22262509/israel-covid-19-vaccinations-serious-illness-decline.

[59] Worldometers. Israel COVID—Worldometer. https://www.worldometers.info/coronavirus/country/israel.

[60] Open Access Government Israel’s Vaccine Data Suggests that Pfizer is Performing at the Expected 95%. https://www.openaccessgovernment.org/israels-vaccine-data/102261/.

[61] Washington Post. Israel Moves to Head of Vaccine Queue, Offering Pfizer Access to Country’s Healthcare Database. https://washingtonpost.com/world/middle_east/israel-pfizer-coronavirus-vaccine-privacy/2021/01/27/b9773c80-5f4d-11eb-a177-7765f29a9524_story.html.

[62] Times of Israel. Poll Shows Public Less Critical of Government’s Response to COVID; Yamina Slips. https://www.timesofisrael.com/poll-shows-public-less-critical-of-governments-response-to-covid-yamina-slips/.

[63] Times of Israel. Some 25% of Israelis Who Haven’t Vaccinated Have No Intention of Doing So. https://www.timesofisrael.com/poll-some-25-of-israelis-who-havent-vaccinated-have-no-intention-of-doing-so.

[64] An Overview of Israel’s Universal Health Care System. https://ldi.upenn.edu/our-work/research-updates/an-overview-of-israels-universal-health-care-system.

[65] US News. Israel, UK, Among Top Performers for Coronavirus Vaccinations. https://usnews.com/news/best-countries/articles/2021-02-04/supply-distribution-logistics-key-to-countries-covid-19-vaccination-efforts.

[66] Adini B., Goldberg A., Cohen R., Laor D., Bar-Dayan Y. (2012). Evidence-based support for the all-hazards approach to emergency preparedness. Isr. J. Heal. Policy Res..

[67] Berger T., Fogel I., Poles L., Aran A.A., Shental O., Kassirer M. (2015). Implications Drawn from a Military Bioterror Exercise in Israel. Heal. Secur..

[68] Edward-Ekpu U. Navigating the Complexities Around a COVID Vaccine in Africa. https://www.brookings.edu/blog/africa-in-focus/2021/01/25/navigating-the-complexities-around-a-covid-vaccine-in-africa.

[69] Oxfam International Small Group of Rich Nations Have Bought Up More Than Half the Future Supply Of Leading COVID-19 Vaccine Contenders. https://www.oxfam.org/en/press-releases/small-group-rich-nations-have-bought-more-half-future-supply-leading-covid-19.

[70] Business Today. ‘Vaccine nationalism will prolong the pandemic, not shorten it,’ warns WHO. https://www.businesstoday.in/industry/pharma/story/vaccine-nationalism-will-prolong-the-pandemic-not-shorten-it-warns-who-276725-2020-10-26.

[71] The Guardian. WHO: Just 25 Covid Vaccine Doses Administered in Low-Income Countries. https://www.theguardian.com/society/2021/jan/18/who-just-25-covid-vaccine-doses-administered-in-low-income-countries.

[72] VOA News. Nigeria’s Goal: Vaccinate 40% of Population Against COVID-19 This Year. https://www.voanews.com/covid-19-pandemic/nigerias-goal-vaccinate-40-population-against-covid-19-year.

[73] WHO International COVAX Facility. Interim Distribution Forecast. https://www.who.int/docs/default-source/coronaviruse/act-accelerator/covax/covax-interim-distribution-forecast.pdf?sfvrsn=7889475d_5%3E.

[74] Worldometers. Nigeria COVID—Worldometer. https://www.worldometers.info/coronavirus/country/nigeria.

[75] Lazarus J.V., Ratzan S.C., Palayew A., Gostin L.O., Larson H.J., Rabin K., Kimball S., El-Mohandes A. (2020). A global survey of potential acceptance of a COVID-19 vaccine. Nat. Med..

[76] Jegede A.S. (2007). What Led to the Nigerian Boycott of the Polio Vaccination Campaign?. PLoS Med..

[77] WHO Press Release—WHO and UNICEF Congratulate Nigeria on Ending Wild Poliovirus; Call for Strengthening of Routine Immunization. https://www.afro.who.int/news/press-release-who-and-unicef-congratulate-nigeria-ending-wild-poliovirus-call-strengthening.

[78] IFC The Logistical Challenges of Rolling Out a COVID-19 Vaccine. https://www.ifc.org/wps/wcm/connect/news_ext_content/ifc_external_corporate_site/news+and+events/news/cm-stories/cmp-s1e7.

[79] Onuah F., Reuters https://www.reuters.com/article/us-health-coronavirus-nigeria-storage-idUSKBN29O253.

[80] Gavi. https://gavi.org/gavi-covax-amc.

[81] Worldometers. Taiwan COVID—Worldometer. https://www.worldometers.info/coronavirus/country/taiwan.

[82] Wang C.J., Ng C., Brook R.H. (2020). Response to COVID-19 in Taiwan. JAMA.

[83] WHO Summary of Probable SARS Cases with Onset of Illness from 1 November 2002 to 31 July 2003. https://www.who.int/publications/m/item/summary-of-probable-sars-cases-with-onset-of-illness-from-1-november-2002-to-31-july-2003.

[84] Taipei Times. Virus Outbreak: Chen Reveals Taiwan’s E-mail to WHO—Taipei Times. http://taipeitimes.com/News/front/archives/2020/04/12/2003734453.

[85] Lee Y. Taiwan’s New ‘Electronic Fence’ for Quarantines Leads Wave of Virus Monitoring. https://www.reuters.com/article/us-health-coronavirus-taiwan-surveillanc-idUSKBN2170SK.

[86] Taiwan Today. CECC Announces Compensation for COVID-19 Quarantine. https://taiwantoday.tw/news.php?unit=2,6,10,15,18&post=173220.

[87] Taiwan News. Taiwan Buys 20 Million Doses of Covid Vaccines, Shots Ready in March. https://www.taiwannews.com.tw/en/news/4090280.

[88] Reuters Taiwan Firms Up AstraZeneca COVID-19 Vaccine, Finds New UK Variant. https://www.reuters.com/article/us-health-coronavirus-taiwan-idUSKBN2940J7.

[89] Biospectrum Asia Taiwan Secures 10 M Doses of AstraZeneca COVID-19 Vaccine. https://www.biospectrumasia.com/news/55/17350/taiwan-secures-10-m-doses-of-astrazeneca-covid-19-vaccine.html.

[90] Taipei Times. COVID-19: Taiwan Secures 200,000 Vaccine Doses. https://www.taipeitimes.com/News/front/archives/2021/02/09/2003752026.

[91] Focus Taiwan Taiwan Unveils Plans to Prioritize COVID-19 Vaccinations. https://focustaiwan.tw/society/202102090023.

[92] Taiwan News. Taiwan to Receive First Batch of COVID Vaccines as Early as Next Week. https://www.taiwannews.com.tw/en/news/4131292.

[93] The Guardian. Why the Delay? The Nations Waiting to See How Covid Vaccinations Unfold. https://www.theguardian.com/world/2021/jan/08/why-the-delay-the-nations-waiting-to-see-how-covid-vaccinations-unfold?fbclid=IwAR3Iw820ZK7Wd_goLcZ_vszi6GcVP8f2RTCsc1SJ0czwQM9o0BKqgNGD45s.

[94] Thomson Foundation Taiwan says to get share of 1.3 million vaccines via COVAX. https://news.trust.org/item/20210204101206-j4zn5.

[95] Focus Taiwan COVAX to Allot 200,000 COVID-19 Vaccine Doses to Taiwan in First Round. https://focustaiwan.tw/society/202102080010.

[96] BBC. Covid-19 Vaccine: First Person Receives Pfizer Jab in UK. https://www.bbc.com/news/uk-55227325.

[97] National Audit Office http://nao.org.uk/covid-19/.

[98] UK COVID-19 Vaccines Delivery Plan. https://www.gov.uk/government/publications/uk-covid-19-vaccines-delivery-plan.

[99] Business Live. The Factories Making AstraZeneca, Pfizer and Other COVID-19 Vaccine in the UK. https://business-live.co.uk/manufacturing/uk-factories-making-astrazeneca-vaccine-19708380.

[100] Novavax Investor Relations—Press Releases & Statements. Novavax COVID-19 Vaccine Demonstrates 89.3% Efficacy in UK Phase 3 Trial. https://ir.novavax.com/news-releases/news-release-details/novavax-covid-19-vaccine-demonstrates-893-efficacy-uk-phase-3.

[101] BBC. Oxford Vaccine: How Did They Make It So Quickly?. https://www.bbc.com/news/health-55041371.

[102] Ewer K.J., Barrett J.R., Belij-Rammerstorfer S., Sharpe H., Makinson R., Morter R., Flaxman A., Wright D., Bellamy D., Bittaye M. (2021). T cell and antibody responses induced by a single dose of ChAdOx1 nCoV-19 (AZD1222) vaccine in a phase 1/2 clinical trial. Nat. Med..

[103] Voysey M., Clemens S.A.C., Madhi S.A., Weckx L.Y., Folegatti P.M., Aley P.K., Angus B., Baillie V.L., Barnabas S.L., Bhorat Q.E. (2020). Safety and efficacy of the ChAdOx1 nCoV-19 vaccine (AZD1222) against SARS-CoV-2: An interim analysis of four randomised controlled trials in Brazil, South Africa, and the UK. Lancet.

[104] Mahase E. (2020). Covid-19: Oxford vaccine could be 59% effective against asymptomatic infections, analysis shows. BMJ.

[105] Biopharma Reporter. Oxford-AstraZeneca COVID-19 Vaccine Granted Emergency Use Authorization in Multiple Countries. https://biopharma-reporter.com/Article/2021/01/06/Oxford-AstraZeneca-COVID-19-vaccine-granted-emergency-use-authorization-in-multiple-countries.

[106] Oxford Mail. What is the EU vs Oxford/AstraZeneca Vaccine Dispute All About?. https://www.oxfordmail.co.uk/news/19051927.eu-vs-oxford-astrazeneca-vaccine-dispute-explained.

[107] Sky News. COVID-19: AstraZeneca Set to Supply Nine Million More Coronavirus Vaccine Doses to EU After Row Over Shortages. https://news.sky.com/story/covid-19-astrazeneca-set-to-supply-nine-million-more-coronavirus-vaccine-doses-to-eu-after-row-over-shortages-12204888.

[108] Baraniuk C. (2021). Covid-19: How the UK vaccine rollout delivered success, so far. BMJ.

[109] BBC. Covid-19: Premier League Stadium Among Latest Vaccine Sites. https://www.bbc.com/news/uk-55929378.

[110] NHS UK. Coronavirus Vaccination Sites. https://www.england.nhs.uk/coronavirus/publication/vaccination-sites.

[111] BBC. Covid Vaccine: How Many People in the UK Have Been Vaccinated So Far?. https://www.bbc.com/news/health-55274833.

[112] The Guardian. Record 343,000 People in UK Receive Covid Vaccine in One Day. https://www.theguardian.com/society/2021/jan/20/record-34300-people-in-uk-receive-covid-vaccine-in-one-day.

[113] Worldometers. UK COVID—Worldometer. https://www.worldometers.info/coronavirus/country/uk.

[114] BBC. Lockdown: Boris Johnson Unveils Plan to End England Restrictions by 21 June. https://www.bbc.com/news/uk-56158405.

[115] Worldometers. US COVID—Worldometer. https://www.worldometers.info/coronavirus/country/us.

[116] NBC News. Volunteers Sought for Next Phase of COVID-19 Vaccine Trial in U.S.; Starting at End of July. https://www.nbcnews.com/health/health-news/covid-19-vaccine-moderna-begin-final-human-trial-end-july-n1233788.

[117] Rosenbaum L. Forbes. How The U.S. Government’s Billion Dollar Bet On Moderna’s Covid-19 Vaccine Paid Off. https://www.forbes.com/sites/leahrosenbaum/2020/12/18/the-feds-risky-billion-dollar-bet-on-moderna-pays-off-as-fda-authorizes-its-covid-19-vaccine.

[118] CDC Information about the Moderna COVID-19 Vaccine. https://www.cdc.gov/coronavirus/2019-ncov/vaccines/different-vaccines/Moderna.html.

[119] Anderson E.J., Rouphael N.G., Widge A.T., Jackson L.A., Roberts P.C., Makhene M., Chappell J.D., Denison M.R., Stevens L.J., Pruijssers A.J. (2020). Safety and Immunogenicity of SARS-CoV-2 mRNA-1273 Vaccine in Older Adults. N. Engl. J. Med..

[120] Baden L.R., El Sahly H.M., Essink B., Kotloff K., Frey S., Novak R., Diemert D., Spector S.A., Rouphael N., Creech C.B. (2021). Efficacy and Safety of the mRNA-1273 SARS-CoV-2 Vaccine. N. Engl. J. Med..

[121] Ledford H. (2020). Moderna COVID vaccine becomes second to get US authorization. Nature.

[122] BBC. Covid: FDA Approves Pfizer Vaccine for Emergency Use in US. https://www.bbc.com/news/world-us-canada-55265477.

[123] Lee M.J.C., CNN Biden Announces Purchase of 200M Vaccine Doses. https://edition.cnn.com/2021/01/26/politics/biden-vaccine-supply-tuesday/index.html.

[124] NPR U.S. Reaches Deal with Pfizer for 100 Million More Vaccine Doses. https://www.npr.org/sections/coronavirus-live-updates/2020/12/23/949541001/u-s-reaches-deal-with-pfizer-for-100-million-more-vaccine-doses.

[125] USA Today. COVID-19 Vaccine Rollout Hasn’t Worked, but Change Is Coming, Vaccine Panel Predict. https://usatoday.com/in-depth/news/health/2021/01/18/experts-slam-chaotic-covid-vaccination-rollout-look-forward-biden/4168188001/.

[126] AP News. States to Receive Initial $3 Billion Infusion for Vaccines. https://apnews.com/article/health-immunizations-coronavirus-pandemic-local-governments-09af97c9ab6a2a3db3f1983b3e467722.

[127] CDC COVID-19 Vaccination. https://www.cdc.gov/coronavirus/2019-ncov/vaccines/index.html.

[128] KFF Estimates of the Initial Priority Population for COVID-19 Vaccination by State. https://kff.org/coronavirus-covid-19/issue-brief/estimates-of-the-initial-priority-population-for-covid-19-vaccination-by-state/.

[129] CBS Debate Rages over Who Should Be Next in Line For Coronavirus Vaccine. https://www.cbsnews.com/news/covid-vaccine-next-in-line-debate.

[130] Brewster J. Here Are the States Breaking From CDC Guidelines On Vaccine Priority. Forbes. https://www.forbes.com/sites/jackbrewster/2020/12/24/here-are-the-states-breaking-from-cdc-guidelines-on-vaccine-priority.

[131] KFF COVID-19 Vaccine Monitor: January 2021. https://kff.org/coronavirus-covid-19/report/kff-covid-19-vaccine-monitor-january-2021/.

[132] Gharpure R., Guo A., Bishnoi C., Patel U., Gifford D., Tippins A., Jaffe A., Shulman E., Stone N., Mungai E. Early COVID-19 First-Dose Vaccination Coverage Among Residents and Staff Members of Skilled Nursing Facilities Participating in the Pharmacy Partnership for Long-Term Care Program—United States, December 2020–January 2021. https://www.cdc.gov/mmwr/volumes/70/wr/mm7005e2.html.

[133] Washington Post. FDA Review Confirms Safety, Efficacy of Single-Shot Johnson & Johnson Coronavirus Vaccine, Especially Against Severe Cases. https://www.washingtonpost.com/health/2021/02/24/johnson-and-johnson-vaccine.

[134] JNJ Johnson & Johnson Announces a Lead Vaccine Candidate for COVID-19; Landmark New Partnership with U.S. Department of Health & Human Services; and Commitment to Supply One Billion Vaccines Worldwide for Emergency Pandemic Use. https://www.jnj.com/johnson-johnson-announces-a-lead-vaccine-candidate-for-covid-19-landmark-new-partnership-with-u-s-department-of-health-human-services-and-commitment-to-supply-one-billion-vaccines-worldwide-for-emergency-pandemic-use.

[135] NBC News. Biden Ups Vaccine Goal to 1.5 million Shots a Day, Says Vaccine to be Widely Available by Spring. https://www.nbcnews.com/politics/white-house/biden-ups-vaccine-goal-1-5-million-shots-day-says-n1255597.

[136] Vox. President Biden’s International Restoration Project Has Begun. https://www.vox.com/2021/1/20/22238609/biden-inauguration-paris-climate-deal-world-health-organization.

[137] UNICEF In the COVID-19 Vaccine Race, We Either Win Together or Lose Together. https://www.unicef.org/press-releases/covid-19-vaccine-race-we-either-win-together-or-lose-together.

